# The Neural Multilineage Differentiation Capacity of Human Neural Precursors from the Umbilical Cord—Ready to Bench for Clinical Trials

**DOI:** 10.3390/membranes12090873

**Published:** 2022-09-09

**Authors:** Daiany de Souza Dobuchak, Priscila Elias Ferreira Stricker, Nathalia Barth de Oliveira, Bassam Felipe Mogharbel, Nádia Nascimento da Rosa, Dilcele Silva Moreira Dziedzic, Ana Carolina Irioda, Katherine Athayde Teixeira de Carvalho

**Affiliations:** The Pelé Pequeno Príncipe Research Institute, Child and Adolescent Health Research & Pequeno Príncipe Faculties, Advanced Therapy and Cellular Biotechnology in Regenerative Medicine Department, Curitiba 80240-020, PR, Brazil

**Keywords:** mesenchymal stem cells, umbilical cord, neural precursor, neurosphere, membrane

## Abstract

Mesenchymal stem cells (MSC) are promising for regenerative medicine as they have a vast differentiation capacity, immunomodulatory properties and can be isolated from different tissues. Among them, the umbilical cord is considered a good source of MSC, as its collection poses no risk to donors and is unrelated to ethical issues. Furthermore, umbilical cord mesenchymal stem cells (UC-MSC) can differentiate into several cell lines, including neural lineages that, in the future, may become an alternative in the treatment of neurodegenerative diseases. This study used a natural functional biopolymer matrix (NFBX) as a membrane to differentiate UC-MSC into neurospheres and their Neural precursors without using neurogenic growth factors or gene transfection. Through the characterization of Neural precursors and differentiated cells, it was possible to demonstrate the broad potential for the differentiation of cells obtained through cultivation on this membrane. To demonstrate these Neural precursors’ potential for future studies in neurodegenerative diseases, the Neural precursors from Wharton’s jelly were differentiated into Schwann cells, oligodendrocytes, cholinergic-, dopaminergic- and GABAergic-like neurons.

## 1. Introduction

Stem cells are the basis of the human organism, giving rise to all cells and tissues in our body. They are characterized as undifferentiated cells with the capacity for self-renewal and are present in several embryonic, fetal and adult tissues. Cell differentiation is determined by epigenetic modifications related to niches in mammalian tissues [1,2].

Mesenchymal stem cells (MSC) can be obtained from fetal tissues such as the amniotic membrane and umbilical cord and adult tissues such as adipose tissue. Currently, MSC represent an essential source of cells for regenerative medicine, as they have a vast differentiation capacity, are easy to obtain, do not express histocompatibility complex molecules and are less related to ethical barriers. In addition, they show promising results in tissue repair in degenerative diseases, mainly due to their ability to migrate to injured sites and secrete cytokines and growth factors that contribute to tissue repair [3,4]. The isolation of MSC can be carried out from several tissues. However, the umbilical cord has been considered a promising source, as its collection does not pose a risk to the mother or the newborn and the isolation process is fast and straightforward. In addition, isolated cells have plasticity similar to embryonic stem cells; however, they have all the advantages and properties of adult stem cells [5,6]. These characteristics make umbilical cord stem cells (UC-MSC) ideal candidates for the treatment of neurodegenerative diseases such as Alzheimer’s [7] and Parkinson’s [8].

The differentiation capacity of UC-MSC is extensive, differentiating into mesodermal, endodermal and ectodermal lineages. Currently, many studies have focused on the ability of UC-MSC to differentiate into cells of neural lineages, as obtaining neural cells is challenging [9]. Neural differentiation can be achieved through several methods, including supplementing the culture medium with neurogenic growth factors, gene transfection or co-culture [10]. However, for the application of these cells in the treatment of diseases, preventive measures are necessary, such as cultivation free from interference and the use of alternative inputs that do not have products of animal origin in their composition (xeno-free), which can cause immune reactions to the receptor when applied to humans—considering the Good Manufacturing Practice (GMP).

In this study, the differentiation capacity of mesenchymal stem cells obtained from Wharton’s jelly (WJ-MSC) and human umbilical cord blood (UCB-MSC) into several neural cells was investigated. The WJ-MSC and UCB-MSC were seeded on the natural functional biopolymer matrix (NFBX) coated culture flasks, without the addition of growth factors or gene transfection. After days, the developed formation of neurospheres in culture was observed by microscopy. The NFBX membrane is a matrix capable of inducing neural differentiation through epigenetic factors related to mechanotransduction, as demonstrated by De Oliveira et al. (2021) [11], and its ability to differentiate UC-MSC into cholinergic-like neurons has already been published [12]. However, the differentiation into other neural cell types such as GABAergic-like neurons, dopaminergic-like neurons, oligodendrocytes or Schwann cells has not been performed.

## 2. Materials and Methods

The Human Ethics Research Committee of Pequeno Príncipe Faculties (CEP-FPP) approved this study, numbered 1,320,984 (2015,11,16). The 12 umbilical cord samples were collected from healthy mothers who signed the Free and Informed Consent Form, and they were processed within 4 h.

### 2.1. Isolation of Wharton’s Jelly Mesenchymal Stem Cells

The isolation of WJ-MSC was performed using the explant method. First, the umbilical cord samples were washed with phosphate buffer saline (PBS) (Sigma-Aldrich, St. Louis, MO, USA) with 3% antibiotic (300 IU/mL penicillin, 0.3 mg/mL streptomycin) (Sigma-Aldrich, St. Louis, MO, USA) to remove blood cells. Next, the cord was cut lengthwise to remove the veins and arteries and was broken into pieces measuring approximately 2 × 2 mm, which were placed onto culture plates and incubated at 37 °C and 5% CO^2^ for 5 min. After initial incubation, a standard culture medium composed of DMEM/Ham-F12 (Sigma-Aldrich, St. Louis, MO, USA), supplemented with 10% fetal bovine serum (FBS) (Sigma-Aldrich, St. Louis, MO, USA) and 1% antibiotic (100 IU/mL penicillin, 0.1 mg/mL streptomycin) (Sigma-Aldrich, St. Louis, MO, USA) was added. The first change to the culture medium was performed 5 days after isolation, and the following changes were performed every 72 h until the cells reached 85% confluence [12,13].

### 2.2. Isolation of Umbilical Cord Blood Mesenchymal Stem Cells

Umbilical cord blood was collected using a heparin-filled syringe and was processed within 24 h. Cells were isolated using the Ficoll-Paque PLUS^®^ density gradient method (GE Healthcare Life Sciences, Chicago, IL, USA). Three parts of blood were added to one part of Ficoll-Paque PLUS^®^ and centrifuged at 22 °C, 1200 rpm for 30 min. After centrifugation, the phase containing the mononuclear cells was removed, and the cells were washed with PBS containing 2% FBS and centrifuged at 1200 rpm for 10 min. The cells were resuspended in 4 mL of the standard culture medium, seeded in 25 cm^2^ culture bottles at a concentration of 4 × 10^5^ cells/cm^2^ and were incubated at 37 °C and 5% CO^2^. The culture medium was changed every 72 h until the cells reached 85% confluence [12,14,15].

### 2.3. Mesenchymal Stem Cells Characterization

All characterizations were performed by the flow cytometry of markers CD29, CD34, CD45, CD73, CD90, CD105 and CD271 (Table S1). The cells were trypsinized and resuspended in 1 mL of PBS containing 5% human albumin—PBS/HA (Sigma-Aldrich^®^, St. Louis, MO, USA). The suspension was then distributed into 5 tubes (200µL/tube) and fluorochrome-conjugated antibodies were added and incubated for 20 min at room temperature (Table 1). Then, 400 µL of PBS/HA was added, the tubes were centrifuged at 1200 rpm for 5 min and the supernatant was discarded. The cells were resuspended in 100 µL of PBS/HA and 10 µL of 7-aminoactinomycin D (7-AAD) (Sigma-Aldrich^®^, St. Louis, MO, USA) was added to tubes 3, 4 and 5, which were incubated for 5 min at room temperature. After incubation, 400 µL of PBS/HA was added to the tubes, which were then homogenized. Samples were acquired on a flow cytometer (FACS Calibur; Becton Dickinson, Franklin Lakes, NJ, USA) and analyzed using Infinity Flow Cytometry software, Version 1.6.0 [16].

The cells were induced to adipogenic, osteogenic and chondrogenic differentiation using the StemPro^®^ Adipogenesis Differentiation Kit, StemPro^®^ Osteogenesis Differentiation Kit and StemPro^®^ Chondrogenesis Differentiation Kit, respectively, to prove the differentiation capacity (ThermoFisher^®^, Waltham, MA, USA). After differentiation, the cells were stained with Oil Red O (adipogenic), Alizarin Red (osteogenic) and Alcian Blue (chondrogenic) (Sigma-Aldrich^®^, St. Louis, MO, USA).

### 2.4. Cell Growth Kinetics

To evaluate the kinetics of cell growth, they were seeded in 24-well plates at an initial concentration of 9 × 10^2^ cells/cm^2^. Cells from Wharton’s jelly were counted for 15 consecutive days, and cells from the umbilical cord blood were counted for 30 consecutive days. The number of viable cells was determined at intervals of 24 h of cultivation.

At the end of the analysis period, the value corresponding to the average number of viable cells every 24 h was obtained, identifying the period of exponential growth.

### 2.5. Production of Neurospheres and Neural Precursors

A natural polyisoprene-based membrane (COLITEX^®^, São Paulo, Brazil) was used to produce neurospheres and their Neural precursors. It was diluted (*v*/*v*) 1:2 in an aqueous solution, placed onto the wells and then exposed to ultraviolet light overnight for sterilization. The culture flasks were coated with the membrane at a rate of 0.5 mL/ cm^2^ [11,12].

After that, this natural functional biopolymer matrix was named the NFBX membrane, and the UC-MSC were seeded at a concentration of 2 × 10^2^ cells/mL and incubated for 20 min at 37 °C and 5% CO_2_. Then, the standard culture medium was added without any neurogenic growth factor. The medium was changed every 72 h until neurospheres formed.

After formation, the neurospheres were mechanically removed from the NFBX membrane with a micropipette and were seeded in membrane-free culture flasks to migrate and expand the neural precursors. The culture medium was changed every 72 h until the precursors reached 85% confluence.

A flow cytometry analysis of Neural precursors was performed for the markers HLA-DR and HLA-ABC related to the histocompatibility complex to demonstrate these cells’ safety for possible future cellular therapies. A cytometry analysis was performed as described above. Furthermore, to test the potential for neuron formation, Neural precursors were submitted to differentiate into different types of neurons as cholinergic-, GABAergic- and dopaminergic-like neurons, oligodendrocyte and Schwann cells. The Neural precursors derived from the WJ-MSC were used for the differentiation into neural cellular types. 

The Neural precursors were seeded at a concentration of 1 × 10^4^ cells/cm^2^ in 24-well plates. Then, the differentiation medium composed of DMEM/Ham-F12 (Sigma-Aldrich^®^, St. Louis, MO, USA) was supplemented with nerve growth factor—NGF (Peprotech^®^, USA), epidermal growth factor—EGF (Peprotech^®^, East Windsor, NJ, USA), basic fibroblast growth factor—bFGF (Peprotech^®^, East Windsor, NJ, USA) and B27 (Gibco^®^ BRL, Life Technologies, Inc., Grand Island, NY, USA) as described by Stricker et al. (2021). The final concentration was maintained for 4 days, after which phenotypic characterization tests were performed [12,17].

### 2.6. Differentiation into Gabaergic-like Neurons

For GABAergic differentiation, Neural precursors were seeded at a concentration of 5 × 10^5^ cells/cm^2^ in 24-well plates and cultured in an induction medium containing DMEM/Ham-F12 (Sigma-Aldrich^®^, St. Louis, MO, USA), 20 ng/mL of EGF (Peprotech^®^, East Windsor, NJ, USA) and 20 ng/mL of bFGF (Peprotech^®^, East Windsor, NJ, USA) for 10 days. The medium was then replaced with a medium composed of DMEM/Ham-F12 (Sigma-Aldrich^®^, St. Louis, MO, USA) supplemented with 10 ng/mL EGF (Peprotech^®^, East Windsor, NJ, USA) and 10 ng/mL of all-trans-retinoic acid (Sigma-Aldrich^®^, St. Louis, MO, USA) which was maintained for 7 days. Finally, the maturation medium containing DMEM/Ham-F12 (Sigma-Aldrich^®^, St. Louis, MO, USA), 10 ng/mL EGF (Peprotech^®^, East Windsor, NJ, USA), 5 ng/mL of brain-derived growth factor-BDNF (Peprotech^®^, East Windsor, NJ, USA) and 1µM of cyclic Di-Buthyril AMP (Sigma-Aldrich^®^, St. Louis, MO, USA) was added and maintained for 7 days [18].

### 2.7. Differentiation into Dopaminergic-Like Neurons

First, the Neural precursors were seeded in 24-well plates at a concentration of 10^3^ cells/cm^2^ and incubated for 24 h at 37 °C and 5% CO_2_ with a standard culture medium. After incubation, the culture medium was exchanged for the inducing medium composed of a neurobasal medium (Gibco^®^ BRL, Life Technologies, Inc., Grand Island, NY, USA) supplemented with 0.5% B27 (Gibco^®^ BRL, Life Technologies, Inc., Grand Island, NY, USA), 100 ng/mL of FGF8 (Peprotech^®^, East Windsor, NJ, USA) and 50 ng/mL of bFGF (Peprotech^®^, East Windsor, NJ, USA). The cells were maintained in this medium for 9 days, and no medium exchange was performed during this period. After 9 days, 50 ng/mL of BDNF (Peprotech^®^, East Windsor, NJ, USA) was added to the inducing medium, which was changed after 16 days and kept until day 32 [19].

### 2.8. Differentiation into Oligodendrocytes

Neural precursors were seeded at a concentration of 5 × 10^4^ cells/cm^2^ and the inducing medium composed of a neurobasal medium (Gibco^®^ BRL, Life Technologies, Inc., Grand Island, NY, USA) supplemented with 10 ng/mL of bFGF (Peprotech^®^, East Windsor, NJ, USA) and 10 ng/mL EGF (Peprotech^®^, East Windsor, NJ, USA) was added. Every 3 days of culture, 50% of the medium was replaced with the neurobasal medium supplemented with 10 ng/mL of platelet-derived growth factor—PDGF-AA (Peprotech^®^, East Windsor, NJ, USA), 10 ng/mL of bFGF (Peprotech ^®^, East Windsor, NJ, USA) and 10 ng/mL of Sonic Hedgehog (Peprotech^®^, East Windsor, NJ, USA). After six days of cultivation, the inducing medium was entirely replaced by a fresh inducing medium. Finally, the final differentiation medium was composed of DMEM/Ham-F12 (Sigma-Aldrich^®^, St. Louis, MO, USA) supplemented with 3% FBS (Sigma-Aldrich^®^, St. Louis, MO, USA) and 15 nM of F3 (R&D System^®^, Minneapolis, MN, USA) [20].

### 2.9. Differentiation into Schwann Cells

For induction, Neural precursors were seeded at a concentration of 5 × 10^3^ cells/cm^2^ and were cultured with a standard medium supplemented with 35 ng/mL of all-trans-retinoic acid (Sigma-Aldrich^®^, St. Louis, MO, USA). After 72 h, the cells were washed with PBS (Sigma-Aldrich^®^, St. Louis, MO, USA), and the differentiation medium composed of DMEM/Ham-F12 (Sigma-Aldrich^®^, St. Louis, MO, USA), 5 ng/mL of PDGF-AA (Peprotech^®^, East Windsor, NJ, USA), 10 ng/mL of bFGF (Peprotech^®^, East Windsor, NJ, USA), 10µM of forskolin (Sigma-Aldrich^®^, St. Louis, MO, USA) and 25 ng/mL of heregulin (Peprotech^®^, East Windsor, USA) NJ, USA) was added [21].

### 2.10. Immunocytochemistry

For all immunocytochemical analyses, the protocol described below was used.

The cells were washed 3 times with PBS and fixed with 4% paraformaldehyde for 20 min at room temperature, then washed again with PBS. The cells were permeabilized with a PBS solution containing 3% Triton X-100 (Sigma-Aldrich^®^, St. Louis, MO, USA) and 10% FBS for 5 min at room temperature and, after incubation, they were rewashed with PBS.

The plates were incubated overnight at 4°C with the primary antibodies (Table 2), washed with PBS and incubated with the FITC-conjugated secondary antibodies. The plates were observed using an inverted fluorescence microscopy (Axio Vert A1, Car Zeiss, Oberkochen, Germany) [11,12,19].

### 2.11. Scanning Electron Microscopy

In scanning electron microscopy, neurospheres and some neural subtypes were analyzed. After induction, the cells were washed 3 times with sodium cacodylate (pH 8.5) (Sigma-Aldrich^®^, St. Louis, MO, USA) at 0.1 M, fixed for 2 h with Karnovsky solution and then dehydrated in increasing solutions of alcohol 30%, 50%, 70%, 90% and 100%.

The samples were subjected to the critical drying point (CPD-Balzers union/Baltec, Germany) and metalized with gold (CPD-Balzers union/Baltec, Germany). The cells were visualized under a scanning electron microscope (Jeol JSM—6360 LV, Japan and Jeol JFM—6010, LA) [12].

### 2.12. Qualitative Reverse Transcription-Polymerase Chain Reaction (RT-PCR)

The RNA was extracted using the RNeasy Mini Kit (Qiagen^®^) and was treated with DNAse following the manufacturer’s guidelines—Turbo DNA-free TM (ThermoFisher^®^, Waltham, MA, USA). Complementary DNA (cDNA) was synthesized using High-Capacity cDNA (ThermoFisher^®^, Waltham, MA, USA).

The RT-PCR reaction was performed using a 20 µL system containing the cDNA sample and the Master Mix (Promega^®^, Madison, WI, USA). The primers used for the reactions are described in Table 3. The ACTB gene is a constitutive gene that was used as a control for the technique.

## 3. Results

### 3.1. Isolation of Wharton’s Jelly Mesenchymal Stem Cells

**Figure 1:**
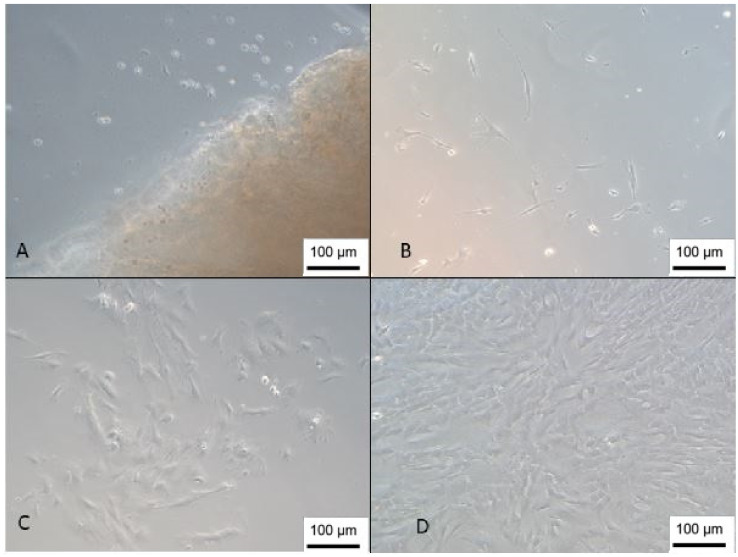
Isolation of the WJ-MSC. (**A**) Cells 1 day after isolation next to a Wharton’s jelly tissue piece; (**B**) day 8; (**C**) day 13; (**D**) cells with 85% confluence (inverted optical microscopy, 100 X). Scale bar, 100 µm.

The isolated cells presented a fibroblastic morphology (Figure 1), as determined by the International Society of Cell Therapy [22], and reached confluence an average of 25 days after isolation by the explant technique, and the proliferation time corroborates the results found in the literature [23]. This technique was chosen because it can obtain a 2.8-fold higher concentration of cells than the enzymatic digestion isolation method [24].

### 3.2. Isolation of Umbilical Cord Blood Mesenchymal Stem Cells

The cells derived from cord blood showed slower proliferation, taking an average of 30 to 60 days to reach confluence. This result differs from another study that reached confluence in an average of 20 days [14]. However, this difference may be related to cell concentration; in the present study, we used a concentration of 1 × 10^7^ cells/cm^2^, while the study by Sibov (2012) [14] used a concentration of 1 × 10^8^ cells/cm^2^.

**Figure 2:**
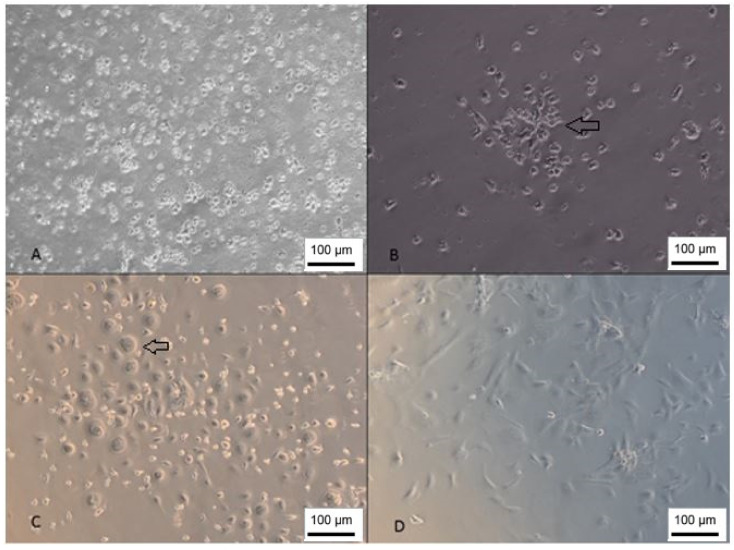
Isolation of UCB-MSC. (**A**) Cells 1 day after isolation; (**B**) day 7—growth in colonies; (**C**) day 15—cells with rounded morphology; (**D**) cells 26 days after isolation (inverted optical microscopy, 100 X). Scale bar, 100 µm.

The isolated cells presented a fibroblastic morphology (Figure 2); however, the isolated population was very heterogeneous, and it is also possible to observe cells with a rounded morphology that, according to Kawasaki-Oyama (2008), have a positive marking for osteoclasts [25]. Furthermore, this population became gradually more homogeneous according to the number of passages.

### 3.3. Mesenchymal Stem Cells Characterization

The flow cytometry analysis revealed that 64% of the WJ-MSC population was positive for the triple labeling of CD73, CD90 and CD105; 71.90% of the population was positive for CD29, 99.95% was negative for CD271 and 99.83% was negative for the hematopoietic markers CD34 and CD45 (histograms are shown in Figures S1 and S2).

As for the cells obtained from the umbilical cord blood, 77.8% were positive for CD73, CD90 and CD73 concerning CD271, 99.99% of the population was negative and 99.86% was negative for CD34 and CD45. These results demonstrate that both Wharton’s-jelly-derived and cord-blood-derived cells meet the minimum criteria established by the International Society for Cell Therapy for the characterization of MSC [22].

**Figure 3:**
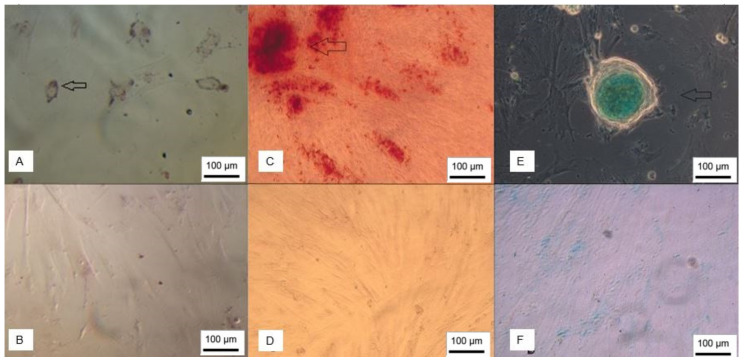
WJ-MSC trilineage differentiation. (**A**) Adipocytes stained with Oil Red O; (**B**) undifferentiated control; (**C**) cells showing mineralization stained with Alizarin Red; (**D**) negative control; (**E**) chondroblasts stained with Alcian Blue; (**F**) undifferentiated control (inverted optical microscopy, ×100). Scale bar, 100 µm.

**Figure 4:**
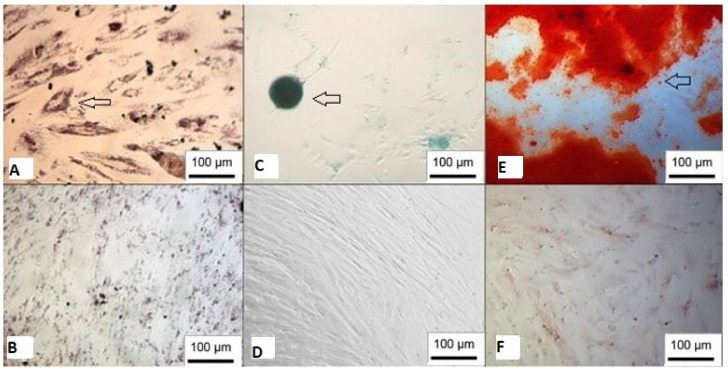
Trilineage differentiation of UCB-MSC. (**A**) Adipocytes stained with Oil Red O; (**B**) undifferentiated control; (**C**) chondroblasts stained with Alcian Blue; (**D**) negative control; (**E**) cells showing mineralization stained with Alizarin Red; (**F**) undifferentiated control (inverted optical microscopy, ×100). Scale bar, 100 µm.

Furthermore, both WJ-MSC and UCB-MSC were able to differentiate into adipogenic, osteogenic and chondrogenic lineages (Figure 3 and Figure 4).

### 3.4. Cell Growth Kinetics

After determining the doubling time, it was possible to observe that the exponential growth of the WJ-MSC was between days 7 and 14, and the doubling time was 5 days (Figure S3). In UCB-MSC, exponential cell growth was observed between days 6 and 19, and the doubling time was 2 days (Figure S4). Despite the doubling time of the UCB-MSC being shorter than that of the WJ-MSC, the cultivation of the former presented more difficulties, mainly about the heterogeneity of the population.

### 3.5. Production of Neurospheres and Neural Precursors

**Figure 5:**
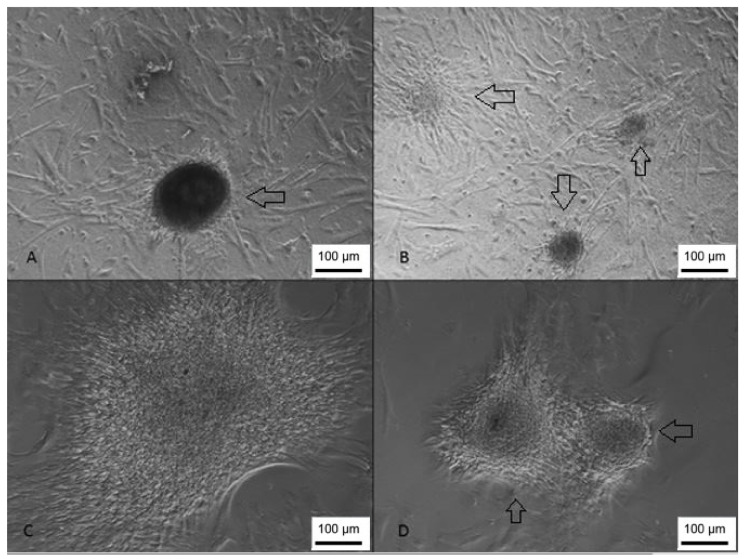
Formation of UCB-MSC-derived neurospheres. (**A**,**B**) Neurospheres visualized at 100 X magnification; (**C**,**D**) neurospheres visualized at 200 X magnification (inverted optical microscopy. Scale bar, 100 µm.

After seeding onto the NFBX membrane, it was possible to observe neurospheres in both WJ-MSC and UCB-MSC. However, the time for neurosphere formation was 2 to 3 days for WJ-MSC and 21 days for UCB-MSC (Figure 5); this difference may be related to the heterogeneity of the population isolated from the umbilical cord blood.

**Figure 6:**
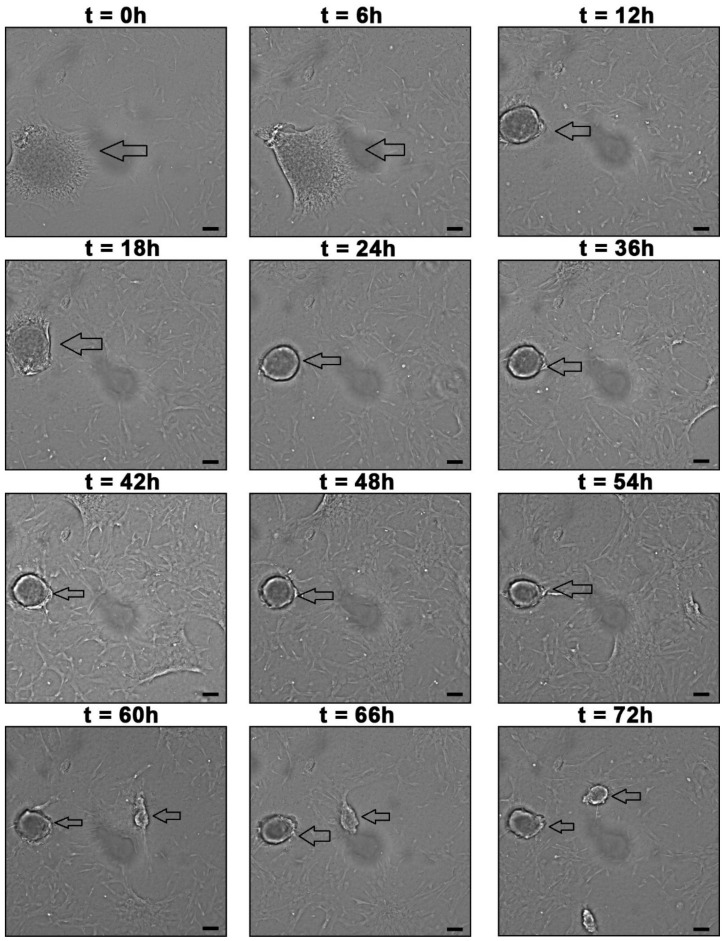
Formation of WJ-MSC-derived neurospheres in 72 h. The arrows point to neurospheres. The images were taken with InCell Analyzer 2000, GE (100 X). Scale bar, 100 µm.

**Figure 7:**
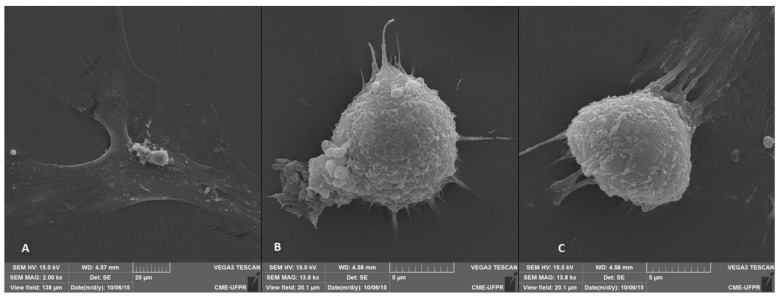
Scanning electron microscopy of WJ-MSC-derived neurospheres. (**A**) undifferentiated WJ-MSC; (**B**,**C**) neurospheres (Jeol JSM—6360 LV, Japan).

To observe the formation of neurospheres, 3 days after seeding the WJ-MSC, the plates were incubated in the InCell Analyzer 2000 to perform photomicrographs every 30 min for 72 h (Figure 6). In addition, scanning electron microscopy was performed (Figure 7)

### 3.6. Neurosphere and Neural Precursors Characterization

**Figure 8:**
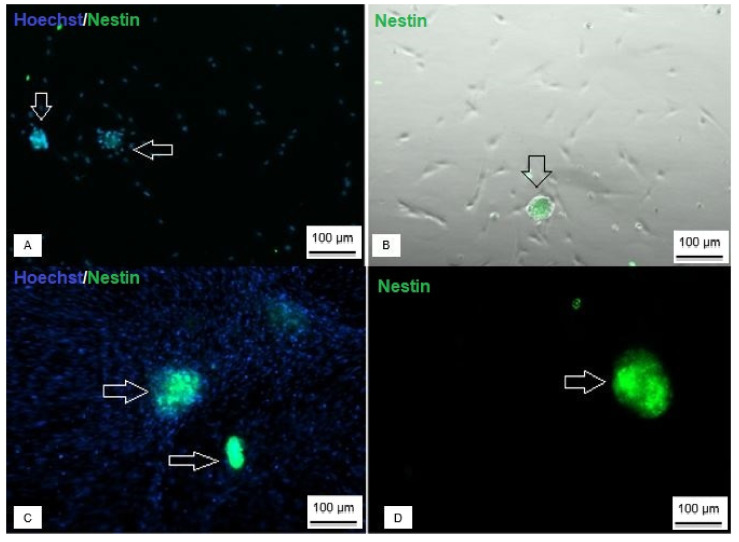
Immunocytochemistry of neurospheres derived from UCB-MSC and WJ-MSC. (**A**) Neurosphere derived from UCB-MSC with nuclear staining (blue) and positive staining for Nestin protein (green); (**B**) phase contrast identifying positive staining for Nestin; (**C**) WJ-MSC-derived neurosphere with nuclear labeling (blue) and positive labeling for Nestin (green); (**D**) positive marking for Nestin (Axio Vert A1, Car Zeiss, Oberkochen, Germany—100 X).

**Figure 9:**
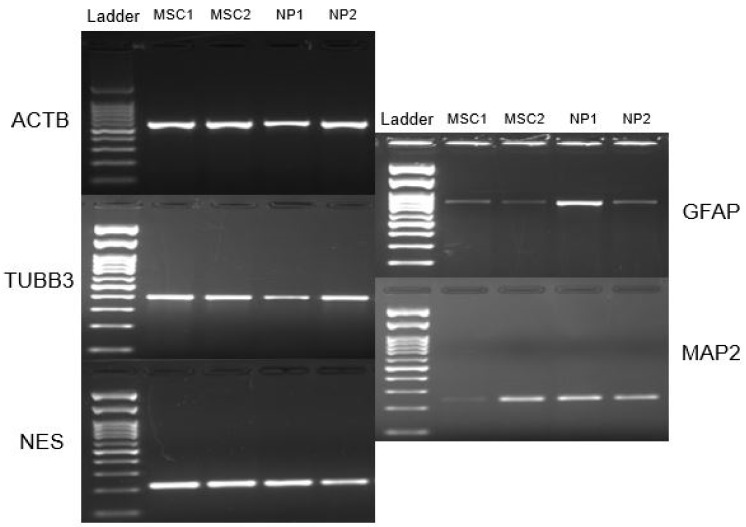
RT-PCR of neurospheres. Note: MSC, and Neural precursors (NP).

Both neurospheres, derived from Wharton’s jelly and those derived from umbilical cord blood, showed positive staining for Nestin neural protein (Figure 8), and regarding the expression of neural genes, it was possible to observe that neurospheres and Neural precursors expressed NES, TUBB3, GFAP and MAP2 genes (Figure 9).

All the genes and proteins analyzed were characteristic of neural cells, so their expression confirms differentiation to the neural lineage.

**Figure 10:**
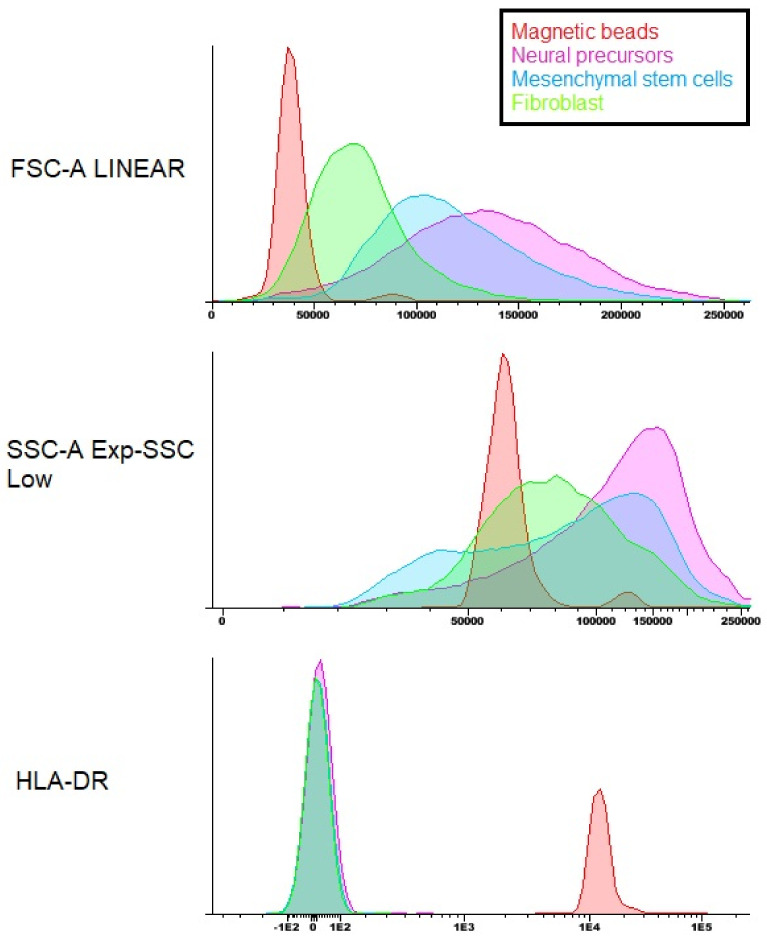
Flow cytometry of the HLA-DR histocompatibility complex molecule. Note: magnetic beads—red; Neural precursor—purple; MSC—blue; fibroblast—green.

**Figure 11:**
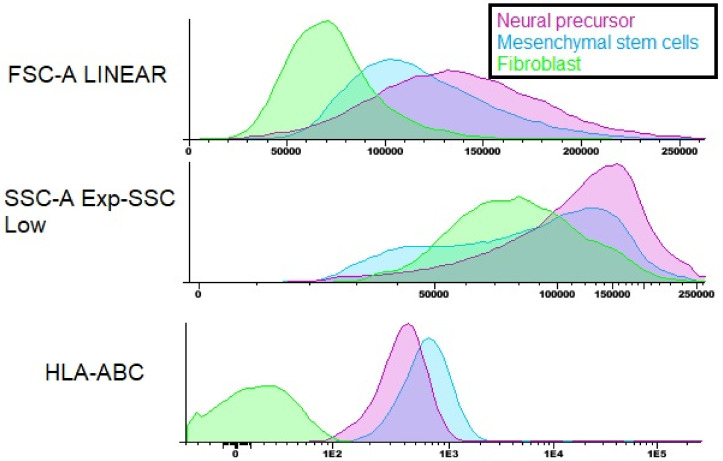
Flow cytometry of the HLA-ABC histocompatibility complex molecule. Note: Neural precursor—purple; MSC—blue; fibroblast—green.

After the flow cytometry of the Neural precursors, it was possible to observe that 70.11% of the population was positive for the markers CD73, CD90 and CD105, 28.98% was positive for CD29, 99.97% was negative for CD271 and 99.68% was negative for the markers CD34 and CD45 hematopoietic. In addition, cytometry was also performed for HLA-DR (Figure 10) and HLA-ABC (Figure 11), which are molecules of the histocompatibility complex. HLA-ABC is present in virtually all nucleated cells in the body, and HLA-DR is present in the membrane of lymphocytes and macrophages and plays an essential role in the transplant rejection response [26].

The cytometry results show that both the WJ-MSC and the Neural precursors showed positive staining for HLA-ABC; however, they were negative for HLA-DR, proving the safety of the use of both cells about the induction of rejection responses to the transplant.

### 3.7. Differentiation into Cholinergic-like Neurons

**Figure 12:**
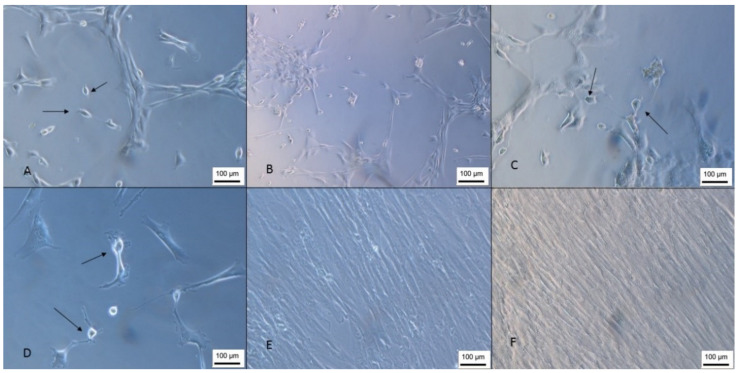
Cholinergic differentiation of Neural precursors. (**A**) Cells 4 days after initiation of induction (100 ×); (**B**) 8 days after induction (100 ×); (**C**) cells 10 days after induction (100 ×); (**D**) 14 days after induction (200 ×); (**E**) WJ-MSC control (100 ×); (**F**) Neural precursor control (100 ×) (inverted optical microscopy). Scale bar, 100 µm.

**Figure 13:**
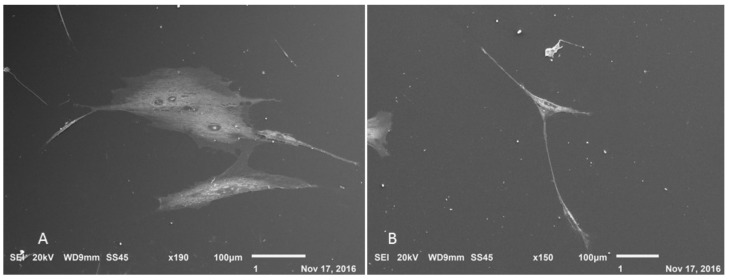
Scanning electron microscopy of cholinergic differentiation. (**A**) Undifferentiated control; (**B**) cell with neural characteristics (Jeol JFM—6010, LA).

After the formation of neurospheres and the induction of cholinergic differentiation, it was possible to observe morphological changes in some cells that showed bipolar characteristics (Figure 12), and during the cultivation, a decrease in the number of cells was observed; however, there was an increase in morphologically altered cells. In addition, visualization with scanning electron microscopy allowed for the observation of cytoplasmic prolongation with a similar morphology to dendrites and possible connections between adjacent cells (Figure 13).

**Figure 14:**
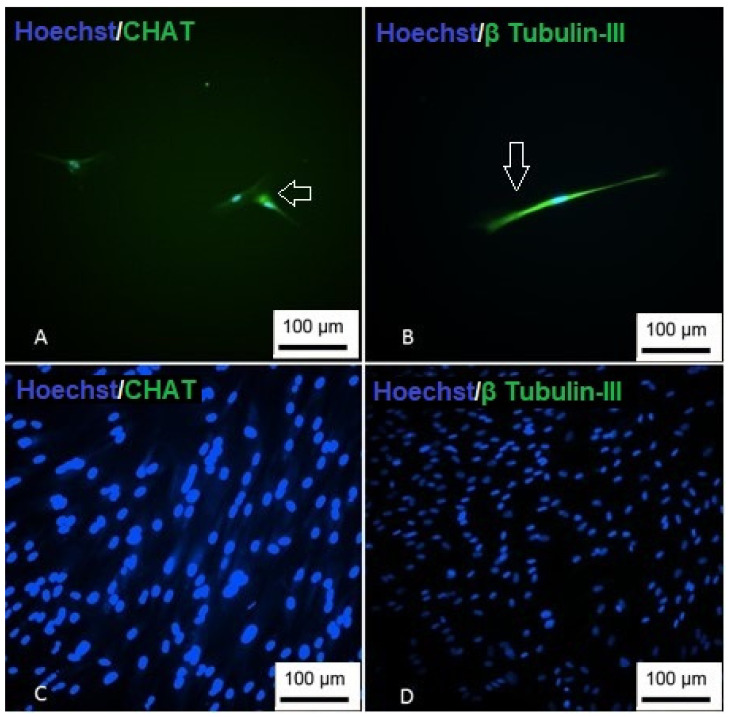
Immunocytochemistry of cholinergic differentiation. (**A**) Cells showing nuclear staining (blue) and positive staining for CHAT (green); (**B**) cells are showing nuclear staining (blue) and positive staining for β Tubulin-III; (**C**) undifferentiated CHAT control; (**D**) undifferentiated β Tubulin-III control (Inverted optical microscopy—100 ×). Scale bar, 100 µm.

To demonstrate the cholinergic differentiation, immunocytochemistry was performed using the CHAT marker and β Tubulin-III (Figure 14). Positive labeling for the proteins demonstrates successful differentiation into cholinergic-like neurons.

### 3.8. Differentiation into Gabaergic-like Neurons

**Figure 15:**
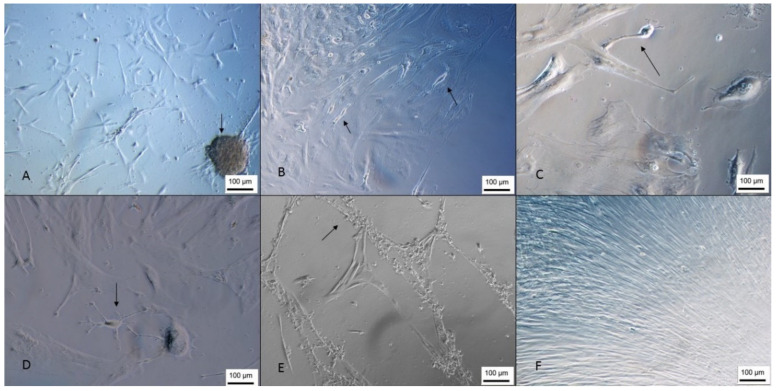
GABAergic differentiation of Neural precursors. (**A**) Cells 4 days after induction (100 ×); (**B**) 8 days after induction (100 ×); (**C**) cells 10 days after induction (200 ×); (**D**) cells 15 days after induction (200 ×); (**E**) 22 days of differentiation (200 ×); (**F**) undifferentiated control—100 × (inverted optical microscopy). Scale bar, 100 µm.

**Figure 16:**
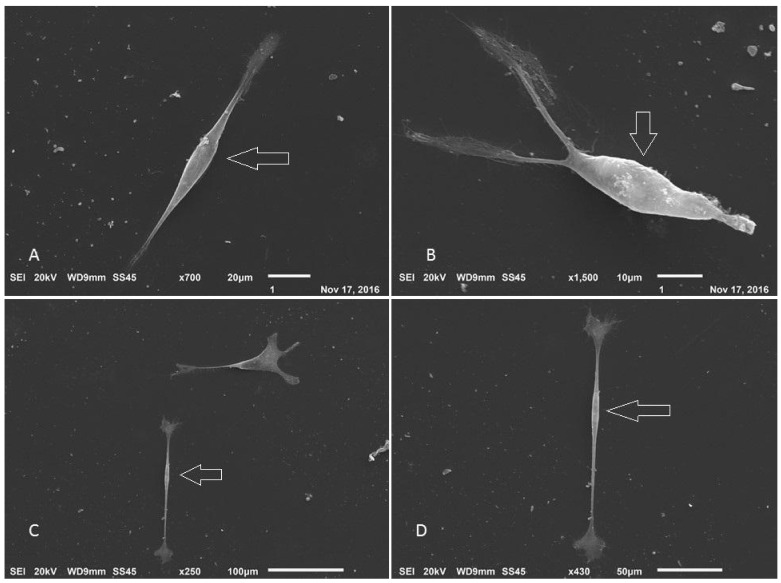
Scanning electron microscopy of GABAergic differentiation. (**A**–**D**) Arrows point to cells with neural characteristics (Jeol JFM—6010, LA).

In the GABAergic differentiation, it was possible to observe the morphological change in the first days after induction, with the cells presenting an elongated and bipolar shape (Figure 15). At the end of differentiation, the formation of vesicles around the cells was observed, and their cytoplasmic processes made connections with adjacent cells, forming a kind of network (Figure 16).

**Figure 17:**
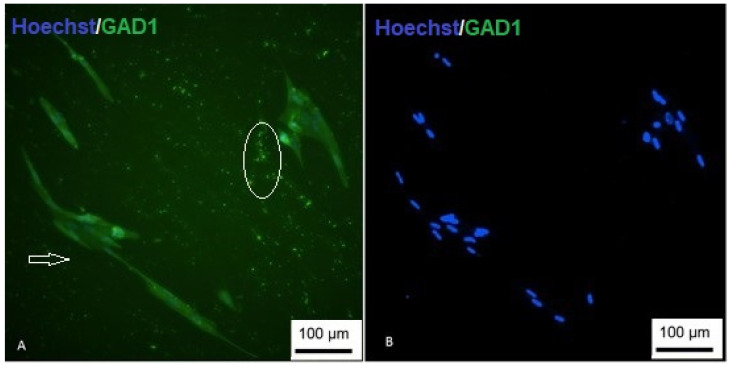
Immunocytochemistry of GABAergic differentiation. (**A**) Cells are showing nuclear staining (blue) and positive staining for GAD1 (green); (**B**) undifferentiated control (inverted optical microscopy—200 ×). Scale bar, 100 µm.

To demonstrate the differentiation, immunocytochemistry was performed using the GAD1 marker. Positive labeling demonstrated successful differentiation (Figure 17).

### 3.9. Differentiation into Dopaminergic-Like Neurons

**Figure 18:**
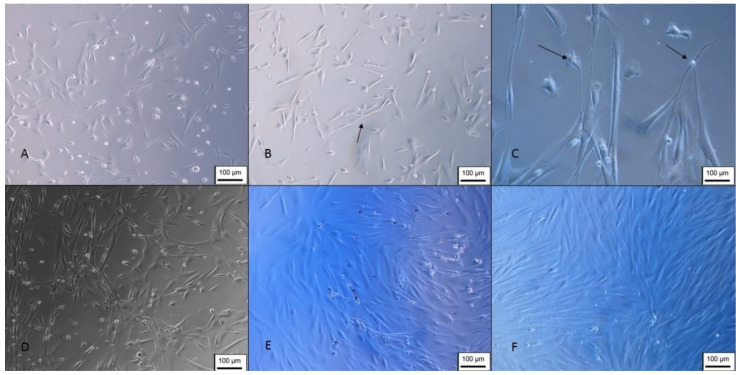
Dopaminergic differentiation of Neural precursors. (**A**) Cells 4 days after induction (100 ×); (**B**) 8 days after induction (100 ×); (**C**) cells 10 days after induction (200 ×); (**D**) cells 15 days after induction (100 ×); (**E**) 20 days of differentiation (100 ×); (**F**) undifferentiated control—100 × (inverted optical microscopy). Scale bar, 100 µm.

In the differentiation into dopaminergic-like neurons, it was possible to observe gradual changes in the cell morphology (Figure 18). Around the tenth day of differentiation, the cells with cytoplasmic processes similar to a dendritic morphology were observed, suggesting connections between the cells. However, there was a decrease in the number of cells after the twentieth day of differentiation.

**Figure 19:**
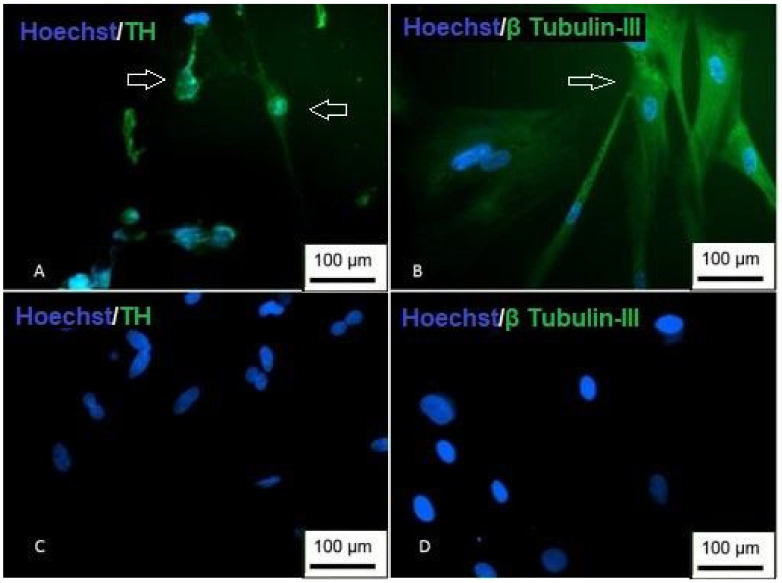
Immunocytochemistry of dopaminergic differentiation. (**A**) Cells are showing nuclear staining (blue) and positive staining for TH (green); (**B**) cells with positive staining for β Tubulin-III; (**C**) undifferentiated TH control; (**D**) undifferentiated β Tubulin-III control (inversion optical microscopy—200 ×). Scale bar, 100 µm.

Immunocytochemistry revealed that the differentiated cells showed positive staining for TH, which is specific for dopamine synthesis in dopaminergic-like neurons. Positive labeling was restricted to cells with morphological alterations, whereas the labeling of the neural cytoskeleton protein, β Tubulin-III, was present in other cells in addition to the differentiated morphology (Figure 19). The morphological changes and the results obtained in the immunocytochemistry demonstrate the dopaminergic differentiation of the Neural precursors.

### 3.10. Differentiation into Oligodendrocytes

**Figure 20:**
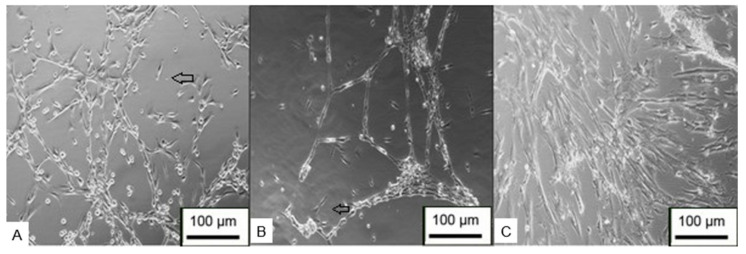
Differentiation in oligodendrocytes from Neural precursors. (**A**) Cells 4 days after induction; (**B**) 8 days after induction; (**C**) undifferentiated control (inverted optical microscopy—100 ×). Scale bar, 100 µm.

In the differentiation into oligodendrocytes, it was possible to observe rapid changes in the cell morphology (Figure 20). With only 4 days of culture in the inducing medium, the cells showed a bipolar process and the formation of connections in a kind of three-dimensional web.

**Figure 21:**
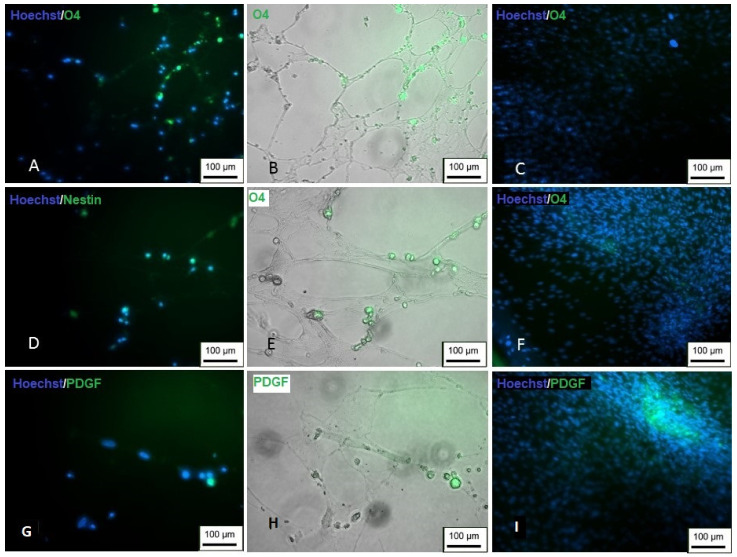
Immunocytochemistry of differentiation in oligodendrocytes. (**A**,**B**) Cells showing positive staining for O4 (green); (**C**) undifferentiated control; (**D**,**E**) positive staining for Nestin (green); (**F**) undifferentiated control; (**G**,**H**) positive staining for PDGF (green); (**I**) undifferentiated control (inversion optical microscopy—200 ×). Scale bar, 100 µm.

The markers Nestin, PDGF and O4 were used to prove the differentiation. Differentiated cells showed positive staining for all the markers used (Figure 21), suggesting the successful differentiation into oligodendrocytes.

### 3.11. Differentiation into Schwann Cells

**Figure 22:**
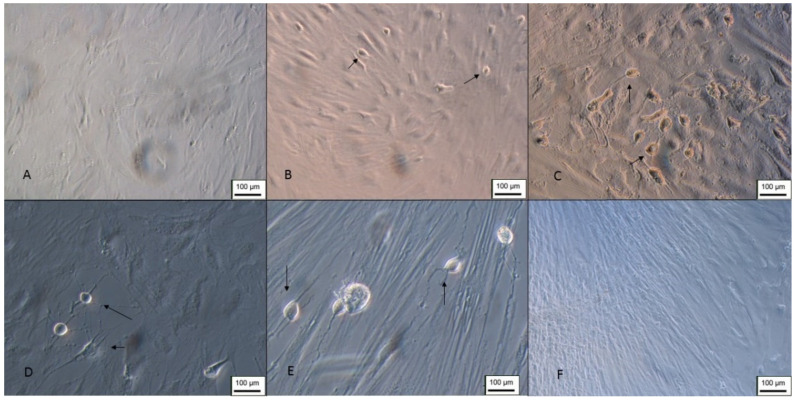
Differentiation in Schwann cells from Neural precursors. (**A**) Cells 4 days after induction (100 ×); (**B**,**C**) 8 days after induction (100 ×); (**D**) cells 10 days after induction (100 ×); (**E**) 10 days after induction (200 ×); (**F**) undifferentiated control—100 X (inverted optical microscopy). Scale bar, 100 µm.

**Figure 23:**
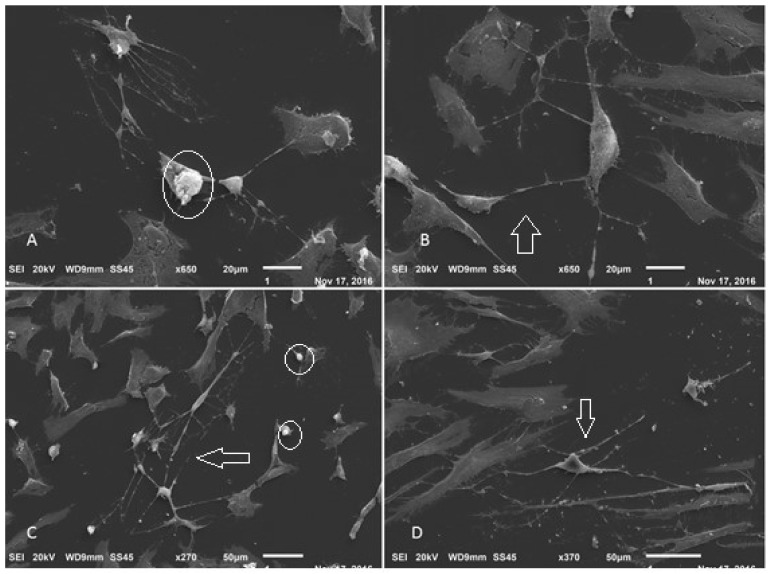
Scanning electron microscopy of Schwann cell differentiation. (**A**–**D**) Arrows point to cells with neural features. Circled details—exocytosis process (Jeol JFM—6010, LA).

After a few days of differentiation, it was possible to observe cells with the typical characteristics of Schwann cells (Figure 22). The cells were elongated and bipolar, with cytoplasmic processes forming connections with adjacent cells. The scanning electron microscopy results demonstrated that the cells were able to form a series of connections, in addition to the characteristic morphology and process of cell excretion by exocytosis, which suggests the production of myelin (Figure 23).

**Figure 24:**
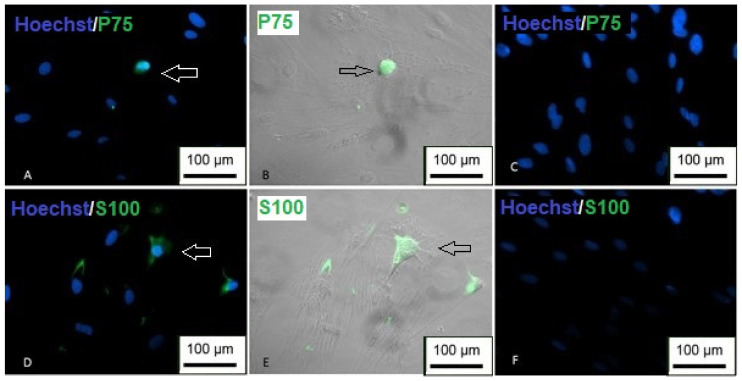
Immunocytochemistry of differentiation in Schwann cells. (**A**,**B**) Cells showing positive staining for P75 (green); (**C**) undifferentiated P75 control; (**D**,**E**) positive labeling for S100 (green); (**F**) undifferentiated S100 control (inverted optical microscopy—200 ×). Scale bar, 100 µm.

To demonstrate the differentiation, immunocytochemistry was performed using the markers P75 and S100. The results showed positive labeling for both proteins, and it is possible to observe that the labeling was restricted only to cells with an altered morphology (Figure 24), indicating the success of differentiation.

## 4. Discussion

MSC have become ideal candidates for regenerative medicine in recent years, mainly due to their immunomodulatory properties, high plasticity, presence in different tissues and lack of ethical barriers that would make their use impractical [27,28]. The umbilical cord has become a promising source of MSC, as its collection poses no risk to donors, is non-invasive and yields a significant number of cells. In addition, UCSC have greater plasticity than the MSC obtained from adult sources such as adipose tissue or bone marrow, and they have a high proliferation rate and an absence of histocompatibility complex molecules, which makes them an important alternative for transplantation studies [29,30,31].

In the present study, MSC were isolated from Wharton’s jelly and human umbilical cord blood. The isolated cells showed MSC characteristics, as demonstrated by flow cytometry, and could differentiate into adipogenic, osteogenic and chondrogenic lineages. These results meet the minimum criteria established for the characterization of SCD by the International Society for Cell Therapy. Then, they demonstrated the growth curve of the WJ-MSC and UCB-MSC with the doubling time of the cells in 5 days and 2 days, respectively. Despite the doubling time of the UCB-MSC being shorter than that of the WJ-MSC, the cultivation of the former was more challenging since the isolated population was more heterogeneous and with the possible presence of osteoclasts. Other studies have reported difficulties in isolating and differentiating umbilical cord blood cells [32,33,34,35]. These difficulties may be related to the low concentration of MSC in the blood and the presence of clots or hemolysis that can occur in blood samples.

Given the neural differentiation ability of UC-MSC, this study aimed to use Neural precursors derived from the neurospheres obtained by cell seeding onto the NFBX membrane to differentiate into various neural types. After cultivation on the NFBX membrane, both WJ-MSC and UCB-MSC were able to form neurospheres with a positive expression of Nestin, a marker of Neural precursors; however, UCB-MSC took approximately 21 days to form neurospheres, while WJ-MSC took approximately 2 to 3 days to form the neurospheres. This difference may be related to the heterogeneity of the UCB-MSC population, as neurospheres are derived from MSC, so the presence of other cells, such as osteoclasts, may have hampered the formation of neurospheres, because they are not able to differentiate in neural cell types. Therefore, for the differentiation in other neural types, we only used the WJ-MSC.

Differentiation into Neural precursors onto the NFBX membrane occurs through mechanotransduction, mediated by YAP and AMOT proteins [11]. The Hippo pathway regulates the activity of these two proteins, and their role in neuronal differentiation/neurogenesis may also be related to the activation of the Wnt/β-catenin pathway [36,37]. Future studies will be important to clarify the role of the Wnt/β-catenin pathway in neural differentiation on the NFBX membrane.

The Neural precursors were characterized from the neurosphere through flow cytometry, immunocytochemistry and RT-PCR. In flow cytometry, it was possible to observe that the Neural precursors derived from MSC still maintained the characteristic markers of MSC, indicating that these cells still had a stem potential. In addition, the expression of histocompatibility complex (HLA-DR) molecules in Neural precursors and MSC was also analyzed. It is already known that one of the reasons why MSC are considered promising candidates for regenerative medicine is their low immunogenicity. One objective was to evaluate whether this characteristic was maintained after cultivation on the NFBX membrane, and if it was possible to observe that even after cultivation in the membrane and the formation of neurospheres, the cells still did not show an HLA-DR expression, demonstrating that the neurospheres’ derived Neural precursors can also be considered promising for regenerative therapies due to their low immunogenicity.

Regarding CD29, it was possible to observe that MSC express the marker more than the population of Neural precursors. It has already been shown that CD29 expression can be reduced in the neurosphere and neural differentiation stages. Furthermore, cells with a low expression of CD29 that also exhibit high staining for CD24 and CD56 can be considered to have a surface antigen pattern consistent with differentiation to neurons or neuroblasts [38]. In the future, other works will be able to evaluate the expression of these other markers for the better characterization of the Neural precursors obtained through cultivation on the NFBX membrane.

An RT-PCR analysis revealed that Neural precursors also express characteristic neural genes such as NES, TUBB3, GFAP and MAP2.

Neural precursors were induced to differentiate into cholinergic-, GABAergic- and dopaminergic-like neurons, oligodendrocyte and Schwann cells. In all of these differentiations, it was possible to observe morphological changes towards that of neural cells. To demonstrate the differentiation, immunocytochemistry was performed for characteristic markers of each neural type, and in all cases, positive marker indicated that the Neural precursors could differentiate into several neural types.

A limitation of this study is that the quantification of the neurotransmitters released by cells was not performed. Therefore, future studies may focus on demonstrating these differentiated cells’ functionality through the quantification of neurotransmitters.

The novelty of this paper is the demonstration of the Neural precursors obtained with the described method: without any addition of induction factors, which were capable of differentiating in various types of nerve cells, among them cholinergic-, dopaminergic- and GABAergic-like neurons, Schwann cells and oligodendrocytes. These differentiations were analyzed retrospectively, and by using the established protocols, it was possible to confirm that those cells were Neural precursor cells with differentiation to more than one type of nerve cell. As demonstrated in our previously published paper, cholinergic differentiation was confirmed [12].

The ability of Neural precursors to differentiate into various types of neural cells is a characteristic that may make these cells promising candidates for the treatment of neurodegenerative diseases. For this reason, it would be interesting if there were minimum criteria for the characterization of Neural precursors as well as the criteria established for MSC. A characteristic cytometry panel, analysis of neural gene expression and neural trilineage differentiation, that is, the differentiation in at least three neural lineages, would be interesting parameters to establish these minimum criteria for Neural precursor characterization.

## 5. Conclusions

In this work, it was possible to conclude that the UC-MSC from both umbilical cord blood and Wharton’s jelly were able to differentiate into Neural precursors through the formation of neurospheres cultured onto the NFBX membrane. Due to the higher yield for the formation of neurospheres from Wharton’s jelly than from umbilical cord blood, differentiations into mature neural types were performed only in neural precursors derived from WJ-MSC. Furthermore, these Neural precursors did not express histocompatibility complex molecules and could differentiate into cholinergic-, GABAergic- and dopaminergic-like neurons, oligodendrocytes and Schwann cells, making them promising cells for future treatments of neurodegenerative diseases.

## Figures and Tables

**Table 1:** Cytometry Panel.

Tube	Content
1	Unmarked cells
2	Isotypic control
3	CD34 FITC/CD271 PE/7-AAD PERCP/CD45 PECY-7
4	CD34 FITC/CD29 PE/7-AAD PERCP/CD45 PECY-7
5	CD73 FITC/CD105 PE/7-AAD PERCP/CD45 PECY-7/CD90 APC

**Table 2:** Antibodies for immunocytochemistry.

Antibodies	Manufactured
Anti-CHAT	Merck Millipore^®^, Burlington, MA, USA
Anti-β Tubulin III	Sigma-Aldrich^®^, St. Louis, MO, USA
Anti-O4	R&D System^®^, Minneapolis, MN, USA
Anti-GAD1/GAD67	R&D System^®^, Minneapolis, MN, USA
Anti-tyrosine hydroxylase (TH)	Sigma-Aldrich^®^, St. Louis, MO, USA
Anti-NGFR p75	Sigma-Aldrich^®^, St. Louis, MO, USA
Anti-S100β	Sigma-Aldrich^®^, St. Louis, MO, USA
Anti-Nestin	Sigma-Aldrich^®^, St. Louis, MO, USA

**Table 3:** Primers for RT-PCR.

Gene	Sequence (5′-3′)	Annealing Temperature	Amplifier Size
MAP2/F	GCTAAATCGTAAGTGAGGGCTG	60 °C	241 bp
MAP2/R	TGGCTCTCTGGCTCTCTAGC	60 °C	241 bp
TUBB3/F	GGAGATCGTGCACATCCAGG	62 °C	385 bp
TUBB3/R	CAGGCAGTCGCAGTTTTCAC	62 °C	385 bp
NES/F	AACAGCGACGGAGGTCTCTA	58 °C	220 bp
NES/R	TTCTCTTGTCCCGCAGACTT	58 °C	220 bp
GFAP/F	CTCACCAAATTCCACCCGCA	60 °C	769 bp
GFAP/R	ACCGCACACAGTACCTGAAG	60 °C	769 bp
ACTB/F	CTGGGACGACATGGAGAAAA	56 °C	564 bp
ACTB/R	AAGGAAGGCTGGAAGAGTGC	56 °C	564 bp

Note: primers obtained from Sigma-Aldrich^®^, St. Louis, MO, USA.

## Data Availability

All data generated or analyzed during this study are included in this submitted article and its supplementary information files.

## Supplementary Materials

The following supporting information can be downloaded at: https://www.mdpi.com/article/10.3390/membranes12090873/s1, Table S1: Markers of flow cytometry; Figure S1: Histogram of the markers CD34, CD271, 7AAD and CD45—WJ-MSC; Figure S2: Histogram of the markers CD73, CD105, 7AAD and CD90—WJ-MSC; Figure S3: Cell growth kinetics of WJ-MSC; Figure S4: Cell growth kinetics of UCB-MSC; Figure S5: Histogram of the markers CD34, CD271, 7AAD and CD45—neural precursors; Figure S6: Histogram of the markers CD73, CD105, 7AAD and CD90—Neural precursors.
