# Supplementary material for: The Neural Multilineage Differentiation Capacity of Human Neural Precursors from the Umbilical Cord—Ready to Bench for Clinical Trials
Source: Membranes (Basel). 2022 Sep 9;12(9):873. doi: 10.3390/membranes12090873 (PMC9500740; doi:10.3390/membranes12090873)
Supplement: Supplementary file 1 [file membranes-12-00873-s001.zip › membranes-1858444-supplementary.pdf]

Supplementary Material

Marker	Fluorochrome	Clone	Manufacturer	Description
CD45	FITC	J33	BD®	It is a membrane protein in lymphocytes and absents in erythrocytes and platelets.
CD34	APC	581	BD®	It is a transmembrane glycoprotein expressed in hematopoietic progenitor cells, vascular endothelium, and some fibroblasts.
CD271	PE	C40-1457	BD®	It is a transmembrane protein found in neuronal axons, Schwann cells, and perineural cells of peripheral nerves. This marker can be found in some epithelial, mesenchymal, and lymphoid tissues.
CD105	PE	266	BD®	It is a membrane protein found in vascular endothelial cells, activated macrophages, and MSC.
CD73	FITC	AD2	BD®	Marker expressed in MSC with enzymatic activity and catalyzes the dephosphorylation of adenosine monophosphate (AMP), converting it to adenosine.
CD29	PE	MAR4	BD®	CD29 is expressed on lymphocytes, monocytes, and granulocytes. It is involved in a variety of cell-cell and cell-matrix interactions.
CD90	APC	5E10	BD®	It is a protein highly expressed in cells capable of being cultured in the long term; therefore, it implies the function of cell-cell adhesion and cell-matrix.

Table S1. Markers of flow cytometry.

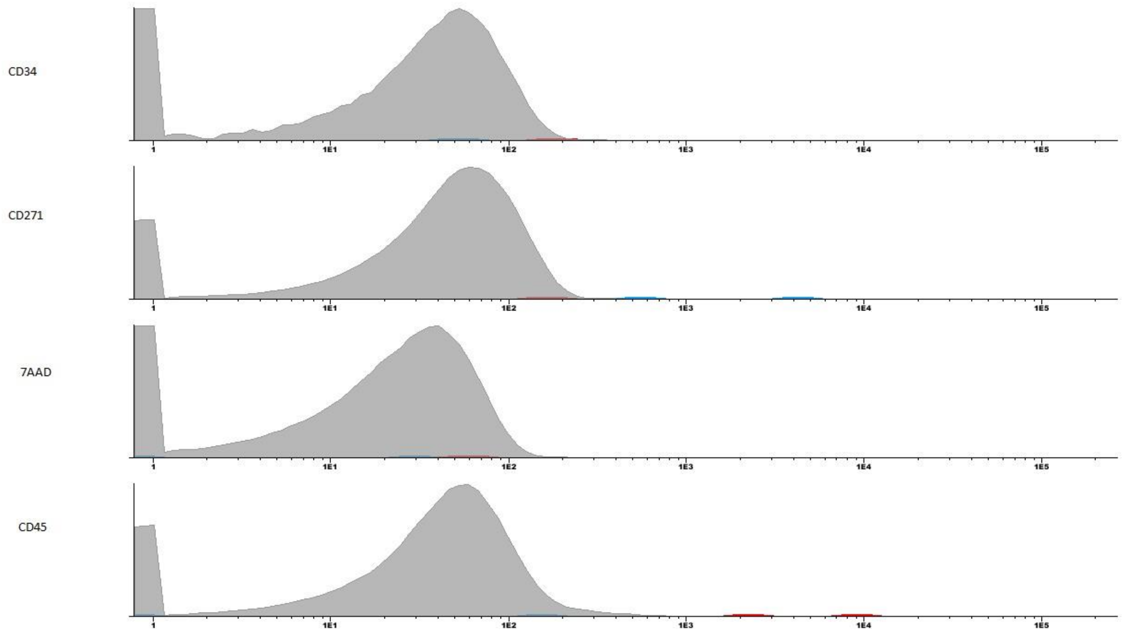

Figure S1. Histogram of the markers CD34, CD271, 7AAD, and CD45 - WJMSC.

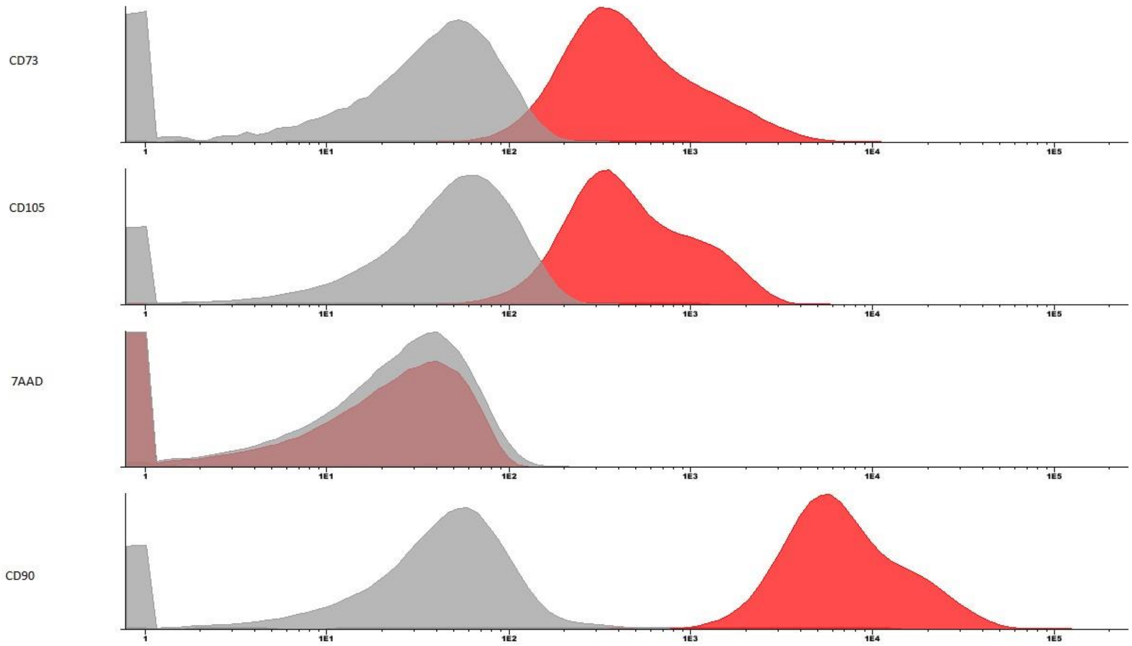

Figure S2. Histogram of the markers CD73, CD105, 7AAD, and CD90 - WJMSC.

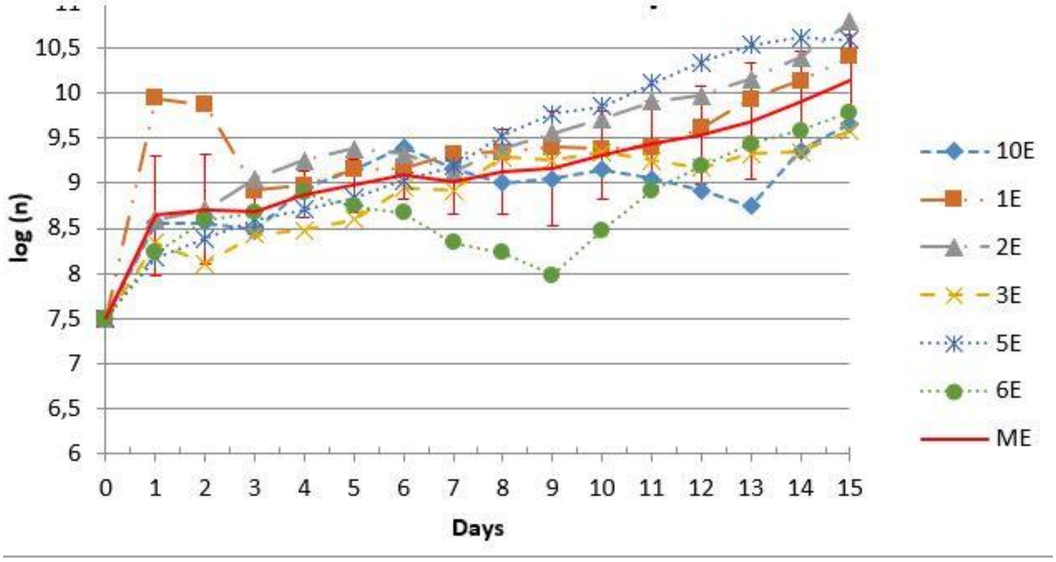

Figure S3. Cell growth kinetics of WJMSC. Note: 1E, 2E, 3E, 5E, 6E, and 10E are the 6 analyzed samples and the samples mean (ME).

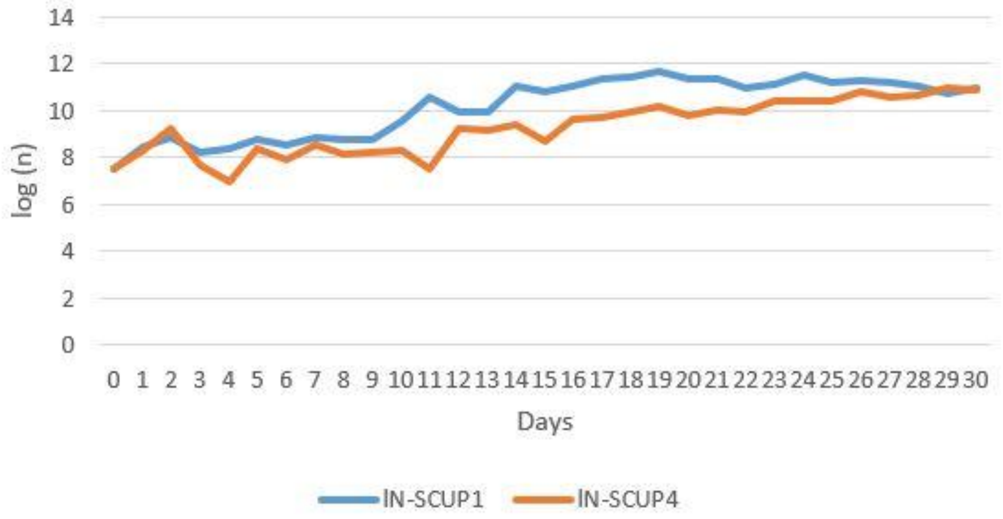

Figure S4. Cell growth kinetics of UCBMSC. Note: IN-SCUP1 and IN-SCUP4 are the 2 analyzed samples.

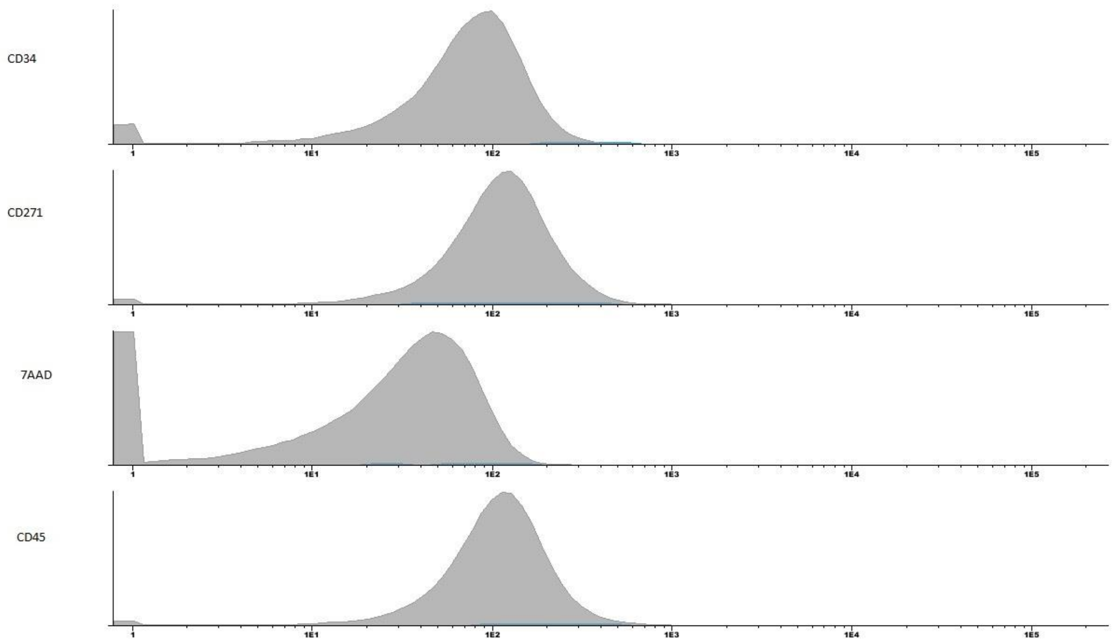

Figure S5. Histogram of the markers CD34, CD271, 7AAD, and CD45 – neural precursors.

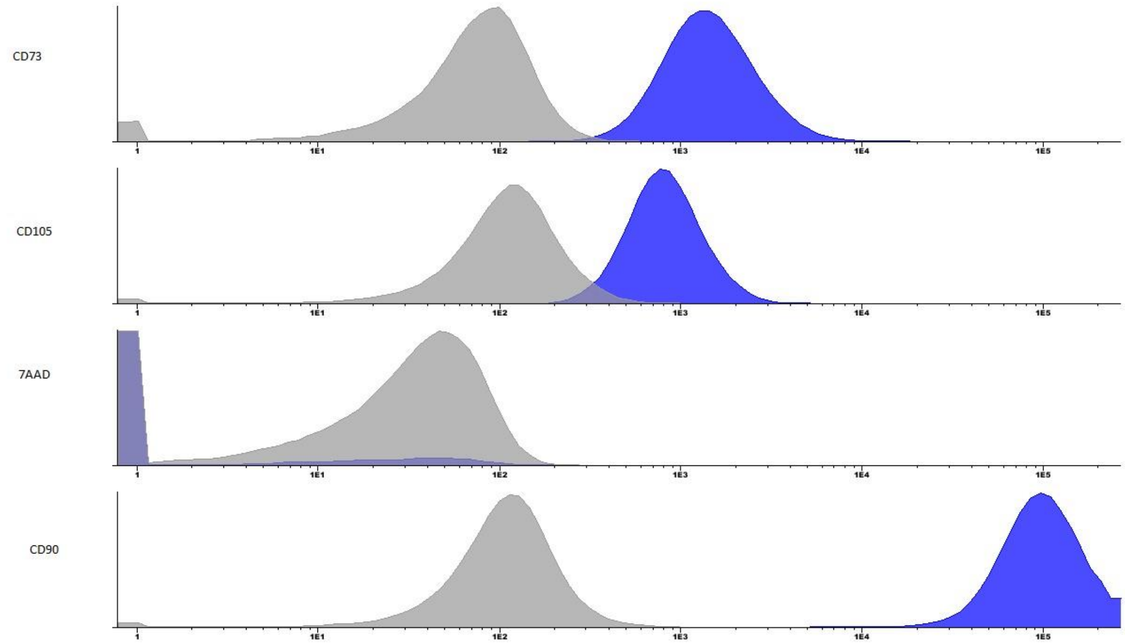

Figure S6. Histogram of the markers CD73, CD105, 7AAD, and CD90 – neural precursors.
